# Level of self-confidence among nurse practitioners in rural public health facilities regarding antimicrobial stewardship programs

**DOI:** 10.1017/ash.2025.178

**Published:** 2025-09-11

**Authors:** Ziyanda Nzayini, Andile Dlungele, Lehlohonolo John Mathibe

**Affiliations:** Division of Pharmacology (Therapeutics), Nelson R. Mandela School of Medicine, University of KwaZulu-Natal, 719 Umbilo Road, Durban 4013, South Africa

## Abstract

**Background:**

Nurse practitioners, especially in remote rural areas in low- and middle-income countries, initiate treatment for numerous conditions including therapy against infections. For a sustained and meaningful reduction in antimicrobial resistance, nurse practitioners should confidently play a greater role as stewards of antibiotic therapy. Therefore, this study investigated the self-confidence level, perceptions, and professional development needs of nurse practitioners as stewards of antibiotic therapy in remote countryside areas in KwaZulu-Natal, South Africa.

**Methods:**

Data collection took place at six healthcare facilities in rural areas in KwaZulu-Natal, South Africa. Questionnaires, with open-ended and 5-point Likert-scale-based items, were distributed to nurse practitioners employed, ie, participants, at the research sites.

**Results:**

One hundred and thirty (n = 130) participants filled and returned questionnaires; 31% (n = 41) and 69% (n = 89) were males and females, respectively. Over 64% (n = 83) of nurse practitioners were not aware of the extent of inappropriate utilization of antibiotics in South Africa, with a median of 3 (interquartile range (IQR 2–3). Over 70% (n = 91) of participants knew that inappropriate utilization of antimicrobials was harmful to patients, with a median of 4 (IQR 3–5). Only 30% (n = 39) of participants felt confident enough to play a meaningful role as stewards of antimicrobial therapy.

**Conclusions:**

There is a need for continuous professional development programs on antimicrobial stewardship to enhance self-confidence among nurse practitioners in rural areas.

## Background

Antimicrobial resistance (AMR) is a global threat.^1^ As a result, in September 2024, the United Nations General Assembly discussed necessary political actions to reduce AMR across the world.^2^ AMR happens when antibiotics are no longer effective against certain types of bacteria, fungi, parasites, or viruses, and the growing emergence of more infectious microorganisms.^3^ Prevalence and the burden of AMR are disproportionately associated with the countries’ levels of income; with low- and middle-income countries (LMICs) being more gravely affected than the high-income countries (HIC).^4^ Although resistant microorganisms can be transmitted from person to person, the main causes include a lack of knowledge and misuse and irrational prescribing of antimicrobials.^5^

Antimicrobial Stewardship (AMS) program is one of the various interventions that have been used to improve utilization, rational prescribing of antibiotics and to reduce and ultimately eradicate AMR.^6^ The main aim of AMS program is to provide an effective, integrated, and multidisciplinary approach to the appropriate use of antibiotic therapy. This program encourages and supports healthcare providers as well as policymakers to utilize antimicrobials carefully and responsibly.

The role played by medical practitioners and pharmacists, especially in the implementation of stewardship programs, is well documented.^7,8^ Recently, Cantudo-Cuenca and colleagues reported that there was over 80% acceptance of pharmacists-led AMS interventions, at healthcare settings with no medical practitioners, particularly with regard to the discontinuation of antibiotics when the duration of therapy was considered excessive.^9^ Medical practitioners, particularly in public healthcare settings, adhere to evidence-based standard treatment guidelines and AMS programs.^10^ However, Hayat and colleagues reported that there was ambivalence regarding the role played by medical practitioners working in public healthcare teaching hospitals in Pakistan about appropriate prescribing of antimicrobials.^11^ While antibiotic stewardship has been introduced to professional nurses, in addition to their traditional roles, they are often faced with numerous institutional and personal barriers. These barriers may relate to their perceived lack of capability as well as poor articulation of professional nurses’ roles in the implementation of AMS policies.^12^ Nurse practitioners are likely to accept positions in rural areas and are easily accessible to the public.^13^ In rural areas in numerous LMICs and in South Africa, authorized professional nurses prescribe and initiate antibiotic therapy as part of their roles.^14,15^ Recently, Anstey Watkins and colleagues reported successfully initiating treatment for various conditions including antimicrobial therapy.^16^ Therefore, for a significant reduction in AMR incidences, nurse practitioners should play a meaningful role as stewards of antibiotic therapy. In contrast to the developed countries,^17^ there is insufficient scientific data on nurse practitioners’ confidence and their educational needs as prescribers and stewards of antibiotics, in rural areas in low- and middle-income countries. There is a marginal difference between self-confidence and confidence. However, the emphasis on self-confidence, as used in this manuscript, is more inward-looking; something similar to self-esteem and self-compassion as in “*Confidence-competence alignment and the role of self-confidence in medical education”* by Gottlieb and colleagues (2022).^18^ Therefore, this study investigated the self-confidence levels and educational needs of nurse practitioners in remote countryside healthcare settings as stewards of antimicrobial therapy. In particular, this study sought to answer the following research questions; (a) what are nurses’ perceptions regarding utilization of antimicrobials? (b) what are nurses’ perceptions and knowledge regarding resistance to antimicrobial therapy? (c) what is the level of self-confidence among nurses regarding what is expected from them as stewards of antimicrobial therapy? and (d) what are their educational needs?

## Methods

### Study design

This was a prospective cross-sectional survey using questionnaires with open-ended and 5-point (ie, 1 = strongly disagree; 2 = disagree; 3 = neutral (don’t know); 4 = agree; 5 = strongly agree) Likert-scale-based items. Permission was granted to adapt and use Wasserman and colleagues’ (2017) questionnaire items to strengthen this study’s questionnaire.^19^

### Study settings

**Figure 1.**
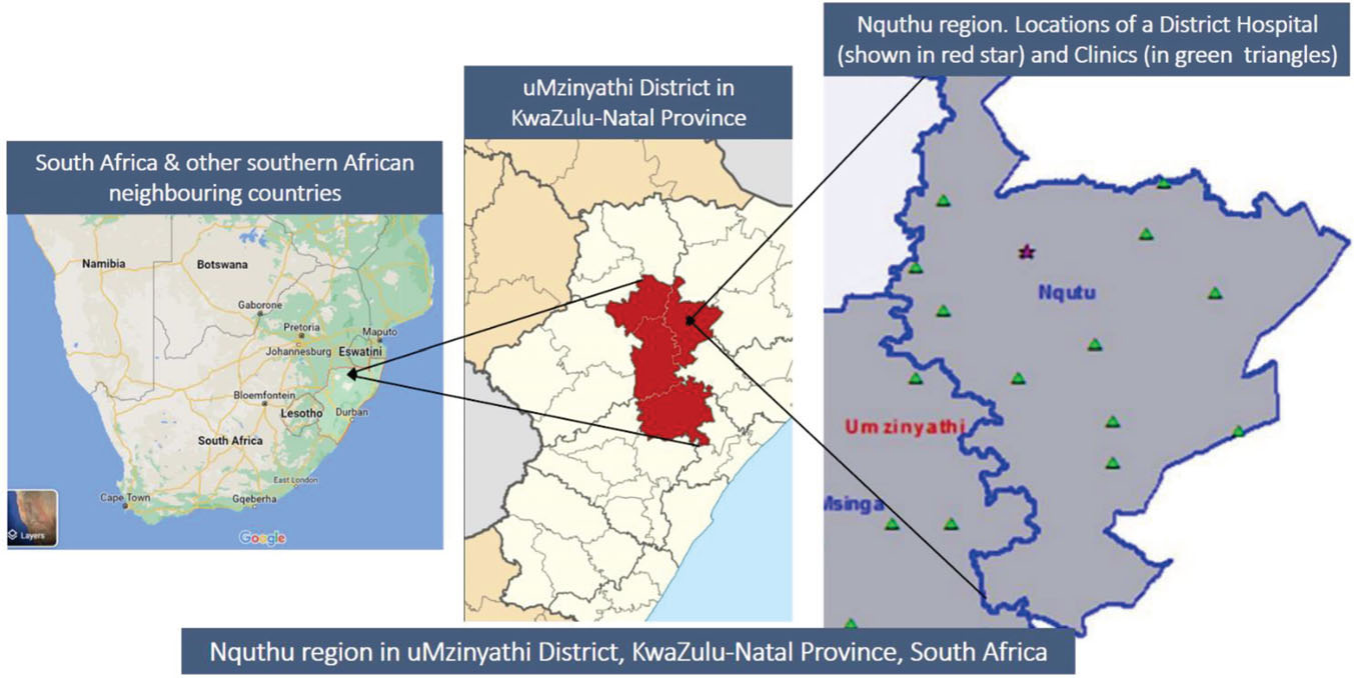
Map of uMzinyathi District in KwaZulu-Nata; Province, South Africa. Modified from Google Maps and HST, 2017; pp 7.^22^

The Nquthu region (or sub-district) in the uMzinyathi District in KwaZulu-Natal Province, South Africa, as depicted in Figure 1, was the main site for this research. This is a deeply rural and the biggest region in the uMzinyathi District with an estimated population size of 178,000 inhabitants.^20^ This area played a significant role in military battles that changed and reformed South Africa in the 1870s.^21^

### Data collection and analysis

Questionnaires were distributed to all categories of nurse practitioners. Namely; professional nurses (with a degree or diploma in nursing—provide comprehensive nursing care and management for the nursing treatment and rehabilitation), staff nurses (with a diploma in nursing—provide general nursing care for the treatment and rehabilitation of individuals and groups), and auxiliary nurses (provide basic nursing care and the primary responsibilities), and enrolled nurses (working under the direct supervision of registered nurse) as defined in the South African Nursing Act of 2005.^23^ Data analysis involved, but was not limited to; participants’ demographics and experience as nurses. Where applicable, statistical significance for categorical variables was tested using the χ^2^ test at a 5% level of significance. Also, the means together with their standard deviations (SDs) and/or 95% confidence intervals (95% CIs) or medians together with their interquartile ranges (IQRs) were calculated. Differences with p-values less than 0.05 were considered significant.

## Results

One hundred and thirty (n =130) participants completed the questionnaires; 69% (n = 89) were females and 31% were males (as depicted in Table 1). The average (the mean, which was similar to the median) age of participants was 40.4 years (sd = 8.4 yr), and the average “period at current position” was 9.2 years (sd = 5.8 yr).

**Table 1.** Demographic characteristics of participants (N = 130). *A level-1 district hospital with 349-bed capacity. **Primary healthcare clinics

Variable		% (n)	Median age in years
**Gender**	Females	69% (n = 89)	42 (IQR 34,0–46,0)
	Males	31% (n = 41)	38 (IQR 33,0–43,0)
**Work settings**	Hospital*	73% (n = 95)	41 (IQR 34,0–45,0)
	Clinics**	27% (n = 35)	38 (IQR 34,0–46,0)
**Categories/Ranks**	Professional Nurses	42% (n = 54)	42 (IQR 33,8–47,0)
	Staff Nurses	29% (n = 37)	42 (IQR 36,6–45,0)
	Enrolled Nurses	25% (n = 33)	38 (IQR 33,0–44,5)
	Auxiliary Nurses	3% (n = 4)	45 (IQR 35,5–49,3)

**Figure 2.**
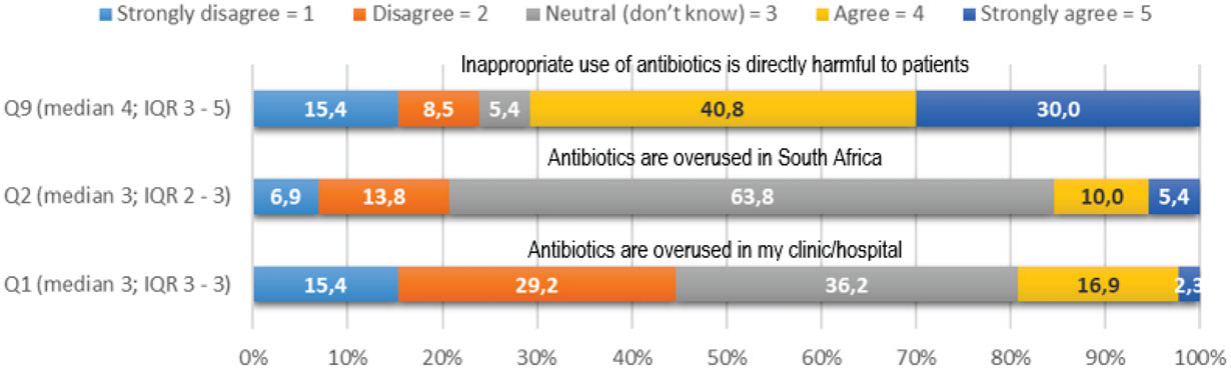
Knowledge regarding appropriate utilization of antimicrobials.

As depicted in Figure 2, 64% (n = 83) of nurse practitioners were not aware of the extent of inappropriate utilization of antibiotics in South Africa, with a median of 3 (IQR 2–3). However, over 70% (n = 91) of participants knew that inappropriate utilization of antimicrobials was harmful to patients, with a medium of 4 (IQR 3–5).

**Figure 3.**
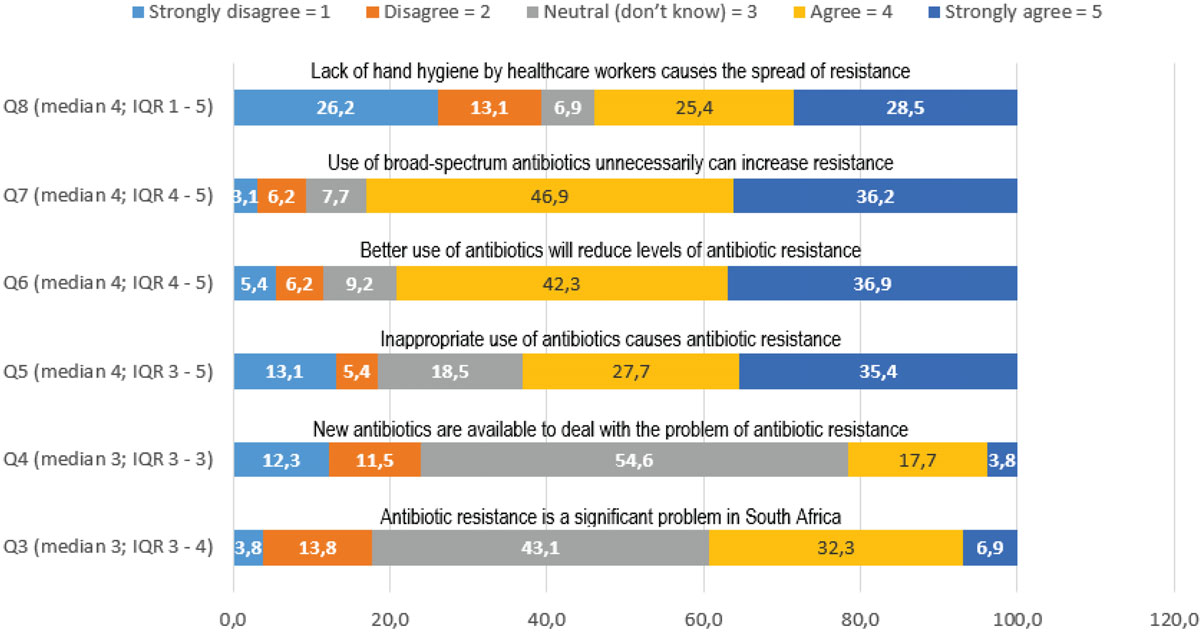
Knowledge regarding antimicrobial resistance.

More than 80% (n = 104) of participants believed that better utilization of antimicrobials reduces resistance to antibiotic therapy. About 83% (n = 108) of nurse practitioners believe that unnecessary use of broad-spectrum antibiotics can increase the risk of antibiotic resistance. Altogether, 55% (n = 72) of the nurse practitioners were unaware whether new antibiotics were available to deal with antibiotic resistance. And about 82% (n = 107) of the nurse practitioners were unaware of the extent of antibiotic resistance in South Africa (Figure 3).

**Figure 4.**
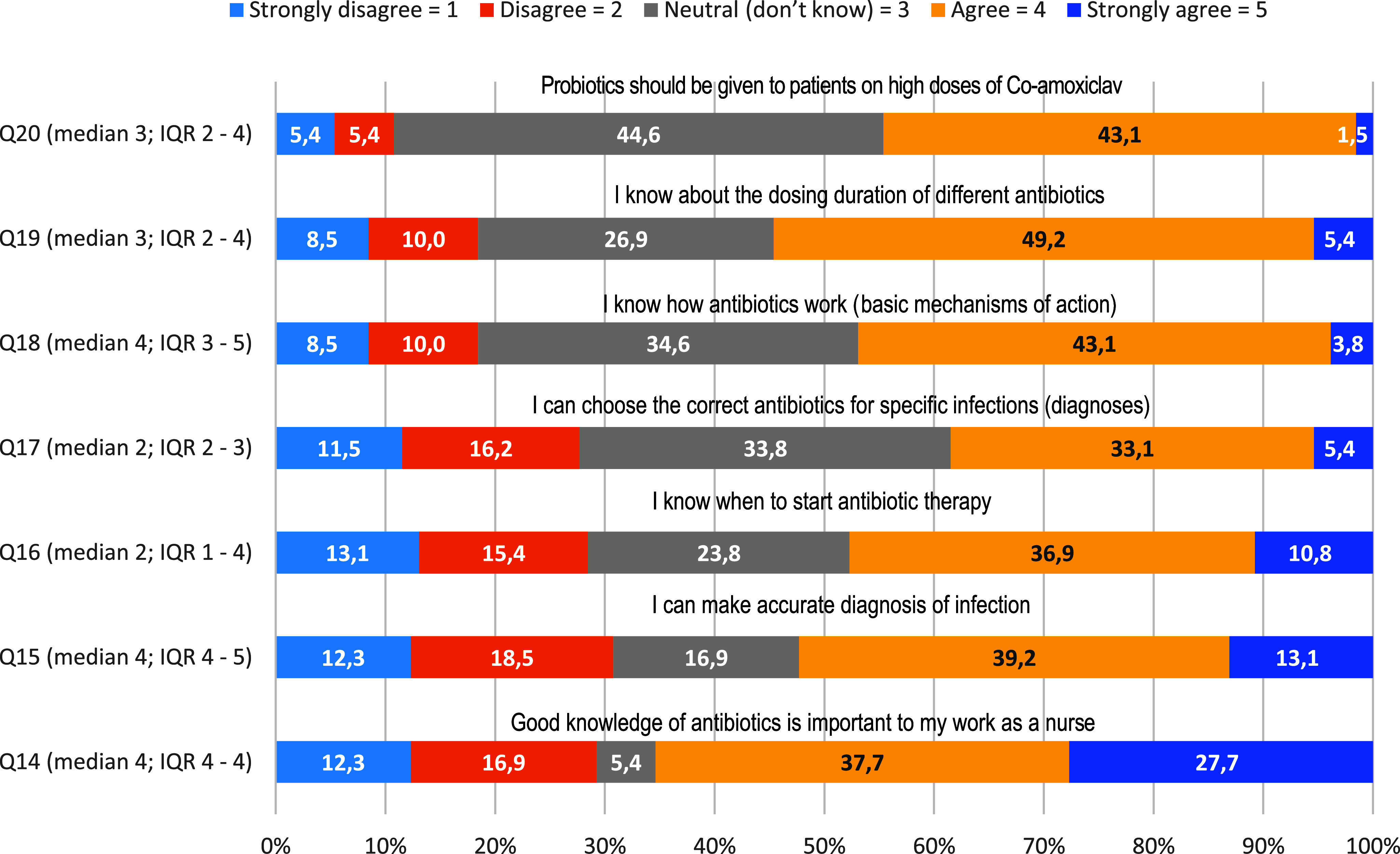
Knowledge regarding the pharmacology of antimicrobials.

As shown in Figure 4, 55% (n = 72) of the nurse practitioners agreed that they knew how to administer antibiotic doses correctly. Fifty-three percent (n = 70) of the nurse practitioners did not know the basic mechanism of antibiotics. Just over 50% (n = 68) of the nurse practitioners mentioned that they knew how to diagnose bacterial infections accurately. About 65% (n = 85) of the nurse practitioners agreed that the knowledge of antibiotics is critical in their profession median 4 (IQR 4-4).

**Figure 5.**
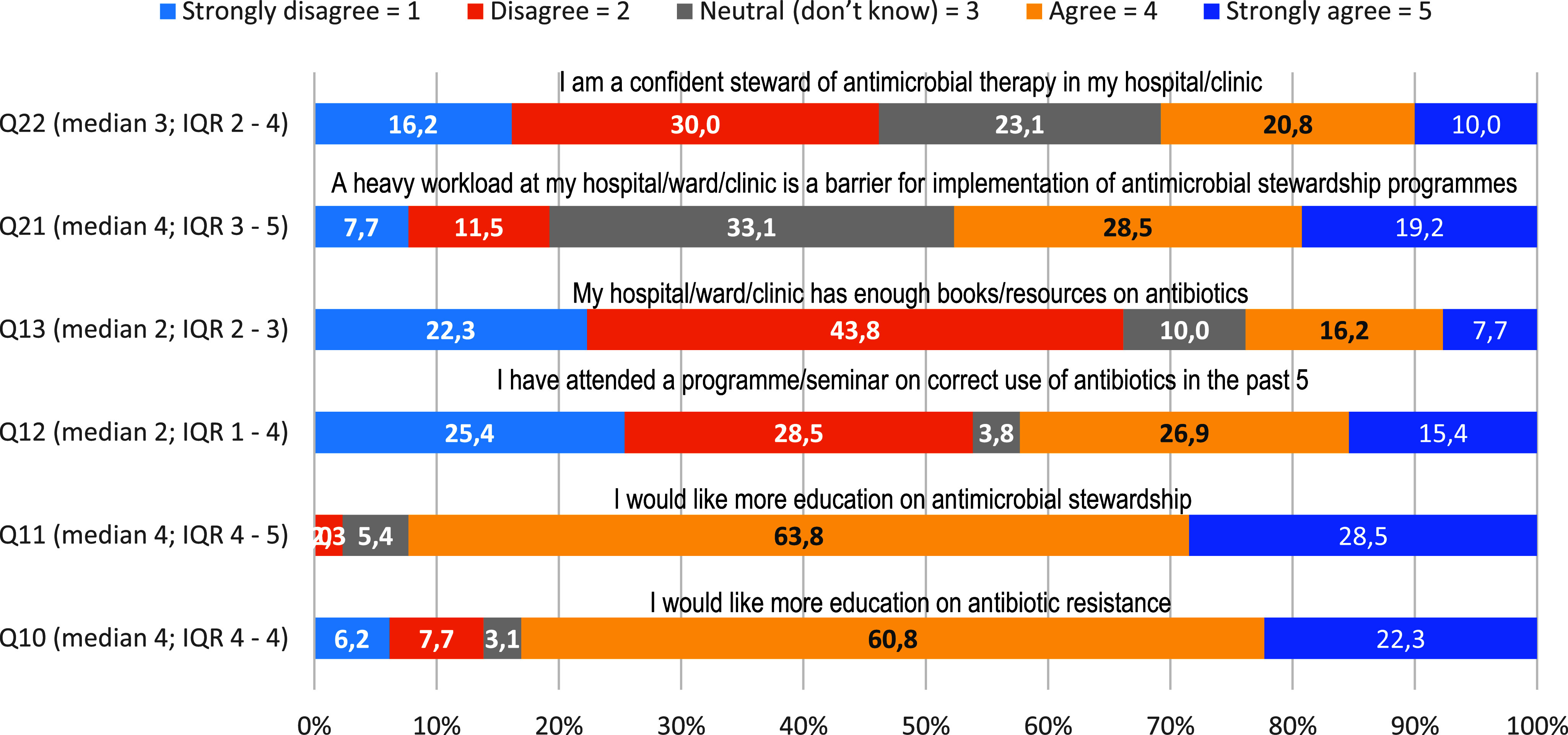
Self-confidence level of nurses as stewards of antimicrobial therapy.

Only 30% (n = 39) of participants felt confident to play a meaningful role as stewards of antimicrobial therapy. As depicted in Figure 5, about 80% (n = 104) and 90% (n = 117) needed more education on AMR and antimicrobial stewardship programs, respectively. Forty-eight percent (n = 72) of the nurse practitioners agreed that the heavy workload is a barrier for implementation of AMS programs.

Over 70% (n = 38) and 51% (n = 51) of professional nurses and staff nurses, respectively, mentioned that they needed help regarding how to choose the correct antibiotic for various infectious diseases. However, the differences were not statistically significant (*P* > 0.05). About 60% (n = 32) professional nurses were not aware/disagreed when asked whether heavy workload, in their facilities, was a barrier to the implementation of an antimicrobial stewardship program; but 56% (n = 22) of staff nurses agreed that heavy workload was a barrier for implementation of antimicrobial programs. As shown in Table 2, 78% (n = 42) and 65% (n = 24) of the professional nurses and staff nurses, respectively, expressed that they had low self-confidence as stewards of antimicrobial therapy.

**Table 2.** Correlations between professional Nurse vs Staff vs Enrolled Nurses; OR = odds ratio; CI = confidence interval; PN = Professional Nurses; SN = Staff Nurses; EN = Enrolled Nurses)

Questions	Ranks	Agree/strongly agree - n	Not sure/disagree/strongly disagree - n	Help needed		Fisher’s exact Test PN vs SN
				Yes %	No %	
I can choose the correct antibiotic (Q17)	Professional Nurses	16	38	70	30	p = 0.08; OR 0.4 (95% CI 0.2 – 1.1)
	Staff Nurses	18	19	51	49	
	Enrolled Nurses	14	15	52	48	
I know about the dosing duration of different antibiotics (Q19)	Professional Nurses	24	30	56	44	p = 0.13; OR 0.5 (95% CI 0.2 – 1.1)
	Staff Nurses	23	14	38	62	
	Enrolled Nurses	19	10	34	66	
Probiotics should be given to patients on high doses of Co-amoxiclav (eg, Augmentin® 1.2 g twice a day); (Q20)	Professional Nurses	21	33	61	39	p = 0.19; OR 0.4 (95% CI 0.2 – 1.3)
	Staff Nurses	20	17	46	54	
	Enrolled Nurses	15	14	48	52	
Heavy workload at my hospital/ward/clinic is a barrier for implementation of antimicrobial stewardship programs (Q21)	Professional Nurses	22	32	41	59	p = 0.14; OR 0.5 (95% CI 0.2 – 1.1)
	Staff Nurses	21	16	56	44	
	Enrolled Nurses	14	15	48	52	
I am a confident steward of antimicrobial therapy in my hospital/clinic (Q22)	Professional Nurses	12	42	78	22	p = 0.20; OR 0.5 (95% CI 0.2 – 1.3)
	Staff Nurses	13	24	65	35	
	Enrolled Nurses	13	16	55	45	

## Discussions

The findings of this study, which was the first to be conducted in deep and remote healthcare settings such as the Nquthu region in KwaZulu-Natal, South Africa, suggest that nurse practitioners have positive attitudes towards their much-needed role as stewards of antimicrobial therapy. However, it was unexpected to discover that self-confidence was low and self-perceived inability to choose the correct antimicrobial therapy was high professional nurses because of the level of their qualifications and experience. Nurse practitioners’ beliefs about their practices and organizational culture were recently identified as factors contributing to perceived low self-confidence as stewards of AMS programs, in metropolitan settings in Missouri, United States.^17^ In this study, however, perceived low self-confidence could have been due to insufficient educational resources and inadequate day-to-day clinical exposure among the most senior nurse practitioners who usually perform managerial roles instead. This is consistent with the findings by Jayaweerasingham and colleagues (2019)—that study reported that self-confidence, among nurse practitioners as stewards of antibiotic therapy, was associated with insufficient routine clinical and prescribing practice.^24^

In support of the findings of our study, Armstrong and colleagues (2015) reported that nursing unit managers spent 25.8% of their time on direct patient care, 16% on hospital administration, 14% on patient administration, 3.6% on education, 13.4% on support and communication, 3.9% on managing stock and equipment, 11.5% on staff management, and 11.8% on miscellaneous activities.^25^ Recently, in support of the findings of our study, Balliram and colleagues (2021) reported low self-confidence level among nurse practitioners regarding prescribing of antibiotics and as stewards of antimicrobials.^26^ In corroboration of the findings of our study, continuous and accessible educational programs have been cited as the best weapons to combat AMR, and to enhance self-confidence among nurse practitioners as the vital stewards of antimicrobial therapy.^27,28^ These interventions are needed both in the public and private healthcare settings.^29^

Reduction of antimicrobial overuse, in public healthcare settings, is one of the critical elements of numerous governments’ national AMR action plans.^30^ Therefore, it was encouraging that a vast majority of nurse practitioners in rural healthcare settings believed that; better utilization of antimicrobials reduces resistance to antibiotic therapy, and that unnecessary use of broad-spectrum antibiotics can increase the risk of antibiotic resistance—as was reported in this study. However, this study found that perceived pressure from patients made it difficult for nurse practitioners to withhold and reduce the utilization of antibiotic therapy. This was in support of Dixon and colleagues’ (2021) findings of a study which was conducted in the urban settings in Zimbabwe.^31^

In our study, nurse practitioners reported that they have experienced, directly and/or indirectly, patients/parents/guardians’ influence on the decision to prescribe antimicrobials. On the contrary, Mathibe & Zwane (2020)^32^ reported that patients/parents/guardians did not influence nurse practitioners’ decisions to prescribe antibiotics. Therefore, there is a need for more research to investigate the impact of patients’ influence and pressure on healthcare workers to prescribe antibiotics.

## Conclusions

There is a need for the formal inclusion of nurse practitioners as an integral part of antimicrobial stewardship programs, globally. Regular use of approved standard treatment guidelines is recommended to enable nurse practitioners to prescribe the correct antibiotics and administer doses correctly for their patients. This will contribute greatly, especially in remote rural areas of low-middle-income countries. In these areas, nurse practitioners are often the only healthcare professionals who are accessible to the vast communities; to diagnose infectious conditions and initiate antibiotic therapy when necessary. Nurse practitioners’ positive attitudes regarding their role in the AMS programs, as reported in our study, will indeed be a stimulus to attenuate their low self-confidence and self-perceived inability to choose the correct antimicrobial therapy. In addition to their inclusion, formal recognition, and appreciation, nurse practitioners require regular and convenient educational programs as well as in-service training activities to enhance their self-confidence as stewards of antimicrobials.^33–35^ More studies are required to investigate the possibility of over or underestimation of self-confidence and the real ability of nurse practitioners to participate meaningfully in the AMS programs.

## Data Availability

Data are available from the corresponding author upon request.
